# The importance of time of day for magnetic body alignment in songbirds

**DOI:** 10.1007/s00359-021-01536-9

**Published:** 2022-01-07

**Authors:** Giuseppe Bianco, Robin Clemens Köhler, Mihaela Ilieva, Susanne Åkesson

**Affiliations:** 1 Centre for Animal Movement Research, Department of Biology, Lund University, Ecology Building, 223 62 Lund, Sweden; 2 Max Planck Institute for Terrestrial Microbiology, 35043 Marburg, Germany; 3 Institute of Biodiversity and Ecosystem Research, Bulgarian Academy of Sciences, 2 Gagarin Str., 1113 Sofia, Bulgaria

**Keywords:** Animal migration, Compass calibration, Compass orientation, Deep neural network, Magnetic compass

## Abstract

Spontaneous magnetic alignment is the simplest known directional response to the geomagnetic field that animals perform. Magnetic alignment is not a goal directed response and its relevance in the context of orientation and navigation has received little attention. Migratory songbirds, long-standing model organisms for studying magnetosensation, have recently been reported to align their body with the geomagnetic field. To explore whether the magnetic alignment behaviour in songbirds is involved in the underlying mechanism for compass calibration, which have been suggested to occur near to sunset, we studied juvenile Eurasian reed warblers (*Acrocephalus scirpaceus*) captured at stopover during their first autumn migration. We kept one group of birds in local daylight conditions and an experimental group under a 2 h delayed sunset. We used an ad hoc machine learning algorithm to track the birds’ body alignment over a 2-week period. Our results show that magnetic body alignment occurs prior to sunset, but shifts to a more northeast–southwest alignment afterwards. Our findings support the hypothesis that body alignment could be associated with how directional celestial and magnetic cues are integrated in the compass of migratory birds.

**Supplementary Information:**

The online version contains supplementary material available at 10.1007/s00359-021-01536-9.

## Introduction

The geomagnetic field has been shown to be a reliable source for orientation and navigation in many animals (Wiltschko and Wiltschko 1995; Lohmann et al. 2007; Mouritsen 2018). Spontaneous magnetic alignment is the simplest known response to the geomagnetic field and, in recent years, has been revealed in a large number of animals, including domestic species such cattle and dogs (Wiltschko 2012; Begall et al. 2013; Burda et al. 2020). The adaptive significance of magnetic body alignment in animals still remains an open question (Begall et al. 2013; Burda et al. 2020). Studies have reported that animals enhance their performance in specific tasks when spontaneously aligning with the geomagnetic field, as in the case of hunting in the red fox *Vulpes vulpes* (Červený et al. 2011) or in case of predator escape in the roe deer *Capreolus capreolus* (Obleser et al. 2016). Only in few cases, magnetic body alignment has been studied in relation to orientation and navigation in vertebrates, such as homing in newts (Phillips et al. 2002; Diego-Rasilla and Phillips 2021) and hunting dogs (Benediktová et al. 2020).

Magnetosensation is a challenging phenomenon to study and the magnetic sensory mechanism is still not completely understood (Phillips et al. 2010; Mouritsen 2018). Magnetic body alignment is particularly difficult to study, because it can be easily affected by other environmental cues such as wind, slopes, landmarks, celestial bodies, olfactory signals, etc., which have extensively been discussed (Begall et al. 2013; Burda et al. 2020). Furthermore, due to the typical bimodal orientation response, particular care should be implemented in handling body alignment data (Begall et al. 2013) and in monitoring the geomagnetic field during data acquisition (Hart et al. 2013a; Bianco et al. 2019a, b). The Emlen-funnel cages (Emlen and Emlen 1966) with which magnetosensation has been extensively studied in migratory songbirds have been shown to be an appropriate method for orientation but not for body alignment studies (Bianco et al. 2016). However, magnetic body alignment can be effectively measured in captive songbirds using larger circular cages and computer vision tracking (Bianco et al. 2016, 2019a).

Birds such as ducks and geese (Hart et al. 2013a), corvids (Pleskač et al. 2017), and pheasant chicks (Čapek et al. 2017) have been reported to align their body with the geomagnetic field. However, to the best of our knowledge, only one study so far has reported that small migratory songbirds align their body with the geomagnetic field during their natural migratory period (Bianco et al. 2019a). Interestingly, these songbirds aligned their body with the magnetic field prevalently during evening hours (Bianco et al. 2019a), that is, in the period that precedes sunset and the onset of nocturnal migratory activity (Berthold 1996; Gwinner 1996; Visser et al. 2010; Bianco et al. 2019b; Åkesson and Helm 2020). It is thought that sunset is the time when compass calibration occurs, because all celestial cues (skylight polarization pattern, sun disk at the horizon, sky glow, stars, etc.) are simultaneously visible in a relatively short time window and the geomagnetic field is more stable (Able and Able 1996; Åkesson et al. 1996, 2002; Cochran et al. 2004; Muheim et al. 2006). However, how the geomagnetic field is integrated with alternative visual cues in songbird migrants remains to be revealed.

In this study, we explored if magnetic body alignment of a nocturnal songbird during its natural autumn migration would occur before or after sunset, and if the timing of body alignment would change in response to a delayed sunset. We implemented a machine learning framework that allowed us to simultaneously monitor the body alignment of two groups of nocturnally migrating Eurasian reed warblers (*Acrocephalus scirpaceus*) in a controlled laboratory setting. One group was experiencing sunset at the local time, while a second group was experiencing a 2 h artificially delayed sunset. With this setting, we could study whether the magnetic body alignment was controlled by the endogenous clock or by the external daylight cue. If magnetic alignment functions as part of the compass calibration process that occurs at sunset, we hypothesized that birds would align their body with the magnetic field prior to and at sunset but not during the dark hours at night. At night, we expect the birds’ bodies to be aligned either randomly or no longer aligned with geomagnetic cardinal axis. We developed a machine learning video analysis method that allowed us to continuously track the birds’ body position around sunset time for 2 weeks. Our results support the hypothesis that magnetic body alignment occurs before and at sunset. They further reveal a possible mechanism involved in the calibration of the biological compasses that could help in the future to interpret cue-conflict studies (Sjöberg and Muheim 2016; Pakhomov and Chernetsov 2020).

## Materials and methods

### Experimental birds and testing facility

First-year migratory reed warblers (*Acrocephalus scirpaceus*) were captured with mist-nets at a stopover site near Stensoffa Ecological Field Station (55°41′ N 13°26′ E) in southwestern Sweden between 4 and 9 September 2019. All birds (*n* = 16) were kept indoors in individual cages until they were moved to the testing facility on 10 September. Birds were divided in 4 groups of 4 individuals each and placed in individual circular cages inside the identical experimental houses built in non-magnetic material and containing 4 cages each (Ilieva et al. 2016). Cages were 500 mm diameter by 700 mm height and equipped with a 3D-printed circular perch to avoid biasing the bird’s body alignment during resting or sleeping (Bianco et al. 2019a). Each experimental house was equipped with a network camera (Axis P1427-LE) that recorded the four cages from above (Ilieva et al. 2018) and a constantly recording magnetometer (Honeywell HMR2300) (Bianco et al. 2019b).

The birds experienced natural light conditions from above, thanks to the semi-transparent roof covering the experimental houses (Åkesson et al. 2021a). To manipulate the time of the sunset, we used an LED lamp (Lumak Pro; 8000 lm luminous flux) with daylight colour temperature in each house. We positioned the lamps to provide diffuse illumination to the inside of the cages to simulate the natural light coming through the roof during daytime (Åkesson et al. 2021a). An electronic timer was set to automatically switch on the lamps in all houses at 17:00 (local time, UTC + 2) and turn the lamps off at 19:30 (local sunset time) in the 2 control houses and 2 h later (21:30) in the 2 experimental houses. At 17:00, the light intensity in the houses was still high and the birds could not notice the light coming from switching the lamps on. Whereas the light intensity provided by the lamps on top of the cages during night-time was comparable with the light intensity measured with an electronic radiometer (IL 1400A, International Light Technologies, Inc., USA) at local sunset in the houses (i.e., around 0.3 mW/cm^2^). Having lamps in both control and experimental houses allowed us: (1) to avoid that the artificial light could affect only the experimental group, and (2) to keep a constant sunset time also for the control group avoiding the effect of the natural quick shortening of day length during autumn at the experimental location for the duration of the experiment as we were interested in the behavioural responses relative to the time of sunset.

We introduced the birds into the cages in the early afternoon of the day before starting the recording to let them familiarise with the new environment. We kept the birds in the cages for a total of 14 days and provided them with fresh food in the form of mealworms and water ad libitum every day at 12:00, local time. All birds were released in the wild at the end of the experiment.

### Measurement of body alignment

**Fig. 1:**
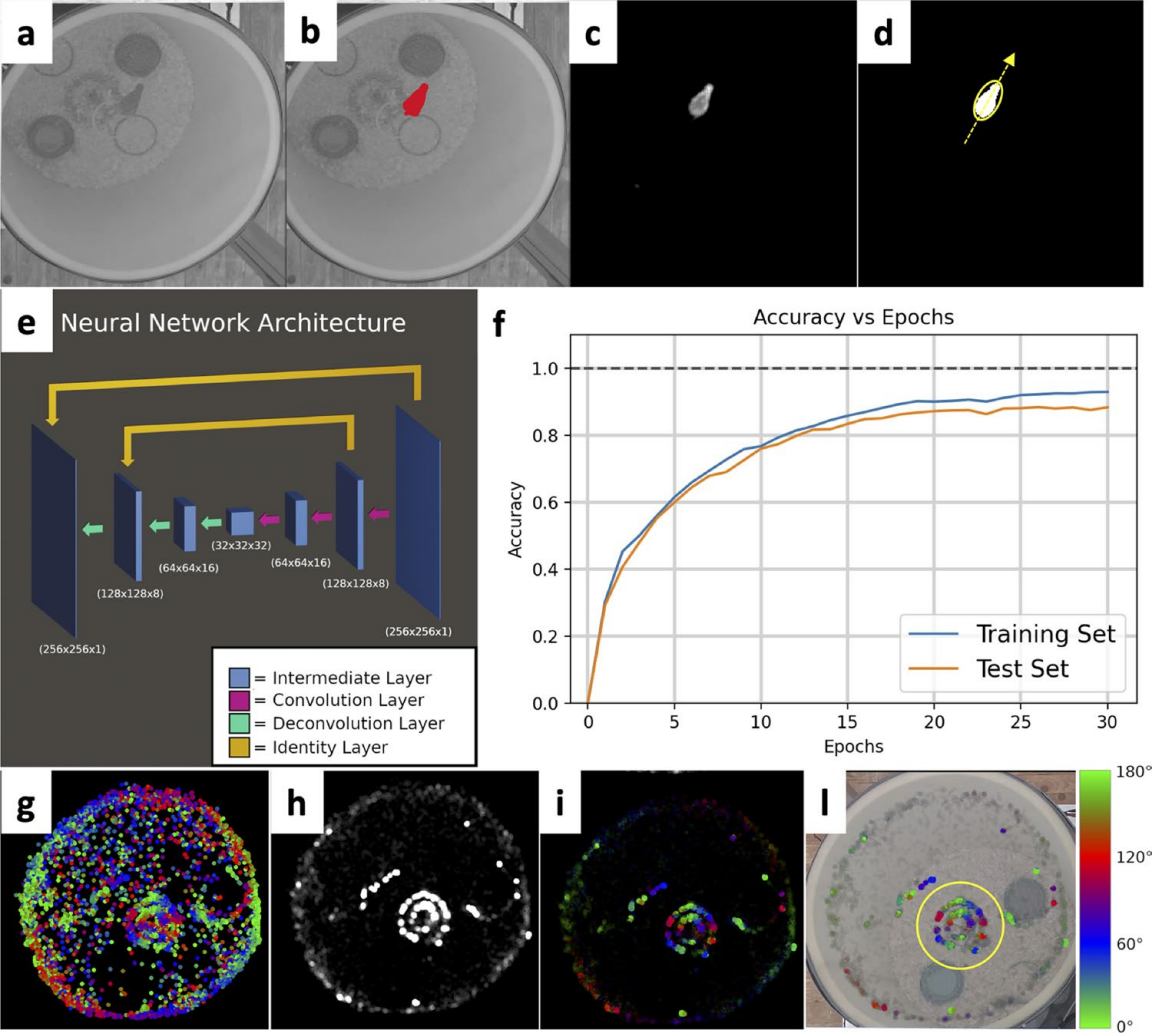
Example of body-axis measurement using a Deep Neural Network (DNN). **a** Frame crop of a single cage showing the bird sitting on the circular perch. **b** Hand-annotated pixels (in red) used to train the DNN. **c** Output of the DNN where brighter intensity corresponds to higher confidence. **d** Body orientation of the bird (yellow arrow) defined as the major axis of the ellipse fitting the bird’s body contour. **e** Architecture of the convolution–deconvolution DNN that takes as input (**a**) and returns (**c**). **f** DDN accuracy (as fraction of correct detected pixels) over the iteration of the training epochs. Both the training set (data used for the training) and test set (data excluded in the training) are shown. **g**–**l** Example of quality check of DNN output. **g** Coordinate of the position of the bird in the cage over the entire recording with colour-coded body alignment direction. **h** Average direction concentration parameter between 0 (black) and 1 (white). **i** Combined information from **g** and **h**. **l** Information in **i** overlayed on the original image together with the circular region of interest (yellow circle) used to define whether the bird is sitting on the circular perch

The birds’ position and their body direction were measured from the video recorded from above the cages using a supervised machine learning algorithm (Fig. 1). We developed a Deep Neural Network (DNN) based on a convolution–deconvolution architecture that is widely used for object detection and is especially efficient for a single object segmentation (Badrinarayanan et al. 2017). The DNN was implemented in Python ver. 3.6 (www.python.org) using the library PyTorch ver. 1.7.1 (Paszke et al. 2019) and consists of 3 convolution layers and 3 deconvolution layers (Fig. 1e). The DNN input is a scaled 256 × 256 image of a single cage that is then transformed into a 32-depth feature map by the convolution layers using a 7 × 7 kernel followed by max pooling (Fig. 1e). The feature map is then upsampled by the deconvolution layers to an output with the same size of the input image (Fig. 1e). To improve prediction of the edge of the bird’s body, we further included 2 identity layers consisting of a convolution of a 1 × 1 kernel to link non-adjacent layers (Yamanaka et al. 2017).

The DNN was trained with the Adam optimization algorithm (Kingma and Ba 2014) using a hand annotated training set of 364 images (Fig. 1b) that was artificially increased by a factor of 8 by flipping and rotating for a total of 2912 images. A combination of mean square error and dice error was used to calculate the training loss. To avoid overfitting, the training process was limited to 30 epochs, after which there was no significant improvement in accuracy (Fig. 1f).

Finally, the body axis of the bird was determined relative to the geomagnetic field between 0° (magnetic North) and 180° (magnetic South) as the direction of the major axis of the ellipse fitting the contour of the bird (Fig. 1d; Bianco et al. 2016, 2019a) using the OpenCV library ver. 3.4.9.31 (Bradski and Kaehler 2008).

### Data analysis

Before statistical analysis, we ensured that the geomagnetic parameters recorded by the magnetometers during the experiment did not show any temporal fluctuations that could affect the behaviour of the birds (Bianco et al. 2019b; Hart et al. 2013b; see example in Supplementary Material, Fig. S1) and that we could use the entire dataset in the analysis outlined below. We then used the information of the bird’s body position and its body axis at 5 frames’ intervals (i.e., 0.83 s) and selected only the body-axis measurements when the bird was sitting on the perch (Fig. 1l) and it was resting (i.e., in the same location for at least 1 min). Such procedure allowed us to exclude all body positions that were indirectly biased by the bird’s movement around the cage (Bianco et al. 2016, 2019a). We analysed body alignment with the procedure of doubling the angles (Batschelet 1981; Begall et al. 2013) using R software version 3.6.3 (R Core Team 2020) and the packages *circular* version 0.4–93 (Agostinelli and Lund 2013) and *bpnreg* version 2.0.1 (Nuñez-Antonio and Gutiérrez-Peña 2014).

To account for autocorrelation in the data caused by periods when the birds kept the same body position for prolonged time, we averaged the body-axis angles in 10-min intervals (*n* = 3383). We grouped the results in 1-h periods by pooling the measurements of individual birds across the entire experimental period and evaluated whether either of the two geomagnetic field axes were included in the 95% confidence interval around the mean of body-axis direction (Batschelet 1981; Bianco et al. 2019a). Pooling data is an accepted procedure for data exploration in orientation and alignment studies when the sample size is small and the number of observations for each individual is comparable (e.g., Hart et al. 2013b; Bianco et al. 2019a). However, to account for the imbalance and non-independence of observations, we then fitted a series of Bayesian circular mixed-effects models to our data using the individual ID as random intercept and tested if daylight (i.e., before and after sunset condition for both control and experimental birds) was the best predictor for the fit of our dataset, how much of the random intercept variance it could explain and whether it confirmed the magnetic alignment seen in the explorative phase. We used the *bpnme* function to build the models and used the two deviance information criterions (DIC, DIC_alt_) and the two version of Watanabe–Akaike information criterions (WAIC_1_ and WAIC_2_) implemented in the *bpnreg* package for model comparison. The mean, mode, SD, and upper and lower highest posterior density interval (HPD) were obtained from the posterior distributions of model estimates (Nuñez-Antonio and Gutiérrez-Peña 2014). The HPD interval is the equivalent of the 95% confidence interval in frequentist statistics for a Bayesian posterior distribution (Gelman et al. 1995) and it was used to infer magnetic body alignment as in the explorative phase described above.

## Results and discussion

**Fig. 2:**
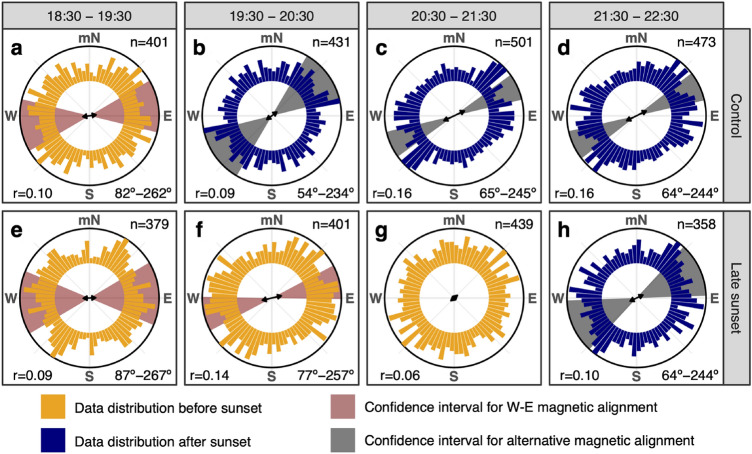
Explorative analysis of body alignment of reed warblers (*Acrocephalus scirpaceus*) measured relative to the magnetic North (mN). Data are reported in 1-h intervals and are relative to a group of eight individuals each kept in individual cages under control and late sunset conditions. Body alignment was measured every 0.83 s by a machine learning algorithm and averaged in 10-min intervals during the course of 2 weeks (*n* = 3383). One group experienced sunset at the natural local time (19:30 UTC + 2; control group) and the second group experienced a 2-h later sunset (21:30; late sunset group). Circular histograms show the normalised angular frequency of body-axis observations and are colour-coded to distinguish whether birds are under daylight (before sunset; yellow) or no daylight (after sunset; dark blue). The double-headed arrows represent the mean vector of axial orientation with length equal to the mean concentration vector *r* (0–1). Number of samples (*n*) and length of the concentration parameter (*r*) are reported for all plots. The axial direction (°) and the 95% confidence interval (shaded areas) are also reported. The confidence interval is reported in red when birds aligned their body orthogonally to the geomagnetic field axis (i.e., the 90°–270° magnetic axis is included in the confidence interval); otherwise, the confidence interval is reported in grey

One hour before local sunset, both the control and the experimental groups were under daylight conditions and reed warblers from both groups were aligning their bodies orthogonally to the geomagnetic field along the W–E magnetic axis (Fig. 2a and e, respectively). The spontaneous alignment of all birds confirms previous results where both diurnal and nocturnal migratory songbirds were aligning their body with the local geomagnetic field (Bianco et al. 2019a). However, in Bianco et al. (2019a), the two nocturnal migrant species chiffchaff (*Phylloscopus collybita*) and European robin (*Erithacus rubecula*) exhibited a bi-axial response during evening hours, whereas the diurnal migrant species the dunnock (*Prunella modularis*) showed an axial response along the N–S magnetic axis (Bianco et al. 2019a, b). Axial orientation (i.e., either along the magnetic axis or orthogonally to it) and bi-axial orientation are equally common in body alignment studies (Begall et al. 2013; Burda et al. 2020) and probably a direct consequence of the mechanism of light-based radical-pair magnetoreception (Phillips et al. 2010; Hore and Mouritsen 2016; Landler et al. 2019). However, it still remains to be explained what mechanism/-s determine the specific body direction in magnetic body alignment in all tested species (e.g., Malkemper et al. 2016).

One hour after local sunset, the control group was no longer exposed to daylight and changed the body alignment in the magnetic NE–SW direction (Fig. 2b), whereas the experimental group still under daylight condition kept its body aligned orthogonally to the geomagnetic field axis (Fig. 2f). The differences in alignment behaviour at this time were recorded simultaneously for control and experimental birds. The difference in body alignment between the two groups, the control experiencing no daylight and the experimental group still exposed to daylight, suggests that the magnetic body alignment along the W–E geomagnetic axis occurs immediately before and at sunset time but not at night. Around sunset: (1) the sun position is still visible, (2) the skylight polarization is stronger near the horizon (Hegedüs et al. 2007), (3) the stars and celestial bodies become again visible (Åkesson et al. 1996), and (4) the geomagnetic parameters become more stable (Skiles 1985). In brief, during a limited period at sunset, all known celestial and magnetic compass cues used by songbirds are simultaneously available and it can be expected that compass calibration occurs around this time (Åkesson et al. 1996; Cochran et al. 2004; Muheim et al. 2006). The spontaneous body alignment with the magnetic field before and at sunset, hence, could be an important but hereto overlooked mechanism to interpret cue-conflict experiments to explain how compass calibration works (Sjöberg and Muheim 2016; Pakhomov and Chernetsov 2020). It should be noted, however, that also the body alignment after sunset can be considered magnetic alignment, since in our experimental setup, birds did not have access to any landmark or celestial cue. However, the body alignment was not coinciding anymore with the W–E geomagnetic axis after the sunset.

During the dark night hours (3 h in total), the control group kept the same magnetic NE–SW body alignment until the end of the tested period (Fig. 2b–d). Such SW-alignment direction (230°–241° when corrected for local magnetic declination) is compatible with the migratory direction expected in the tested species at the experimental location (Fransson et al. 2020). Also, the diurnal migratory dunnocks aligned their body in their expected migratory direction during the morning hours (Bianco et al. 2019a) when this species normally migrates (Dorka 1966; Ilieva et al. 2018). Both observations mentioned above suggest that magnetic body alignment could be involved in selection of the migratory direction, and not only in compass calibration. One possible explanation could be that magnetic body alignment during peak period for migratory activity is a mechanism that Eurasian reed warblers use for sensing magnetic map information (Kishkinev et al. 2015, 2021), that has already been described for newts (Phillips et al. 2002; Diego-Rasilla and Phillips 2021). This behaviour is probably similar to what has been observed in hunting dogs while starting the homing inbound track with a short run aligned with the magnetic axis (Benediktová et al. 2020), or in the desert ants (*Cataglyphis noda*) that align relative to their nest using the earth’s magnetic field during the stereotypical “look back” behaviour during a foraging trip (Fleischmann et al. 2018).

At night-time, the experimental group, experiencing 2 h later sunset, switched to the magnetic NE–SW axis body alignment (Fig. 2h) as it happened for the control group right after local sunset (Fig. 2b). In other words, the experimental birds showed the same alignment behaviour after sunset but delayed by 2 h following the manipulated later sunset time they were experiencing (Fig. 2). Probably, the absence of daylight triggered the switch of body alignment from orthogonal to the geomagnetic field parallel to the expected geographical migratory direction (SW) pointing at the importance of daylight presence/absence as a complementary trigger mechanism for magnetic body alignment behaviour in the tested species.

Reed warblers in the experimental group did not show any preferred body alignment direction 1 h before the simulated sunset (20:30–21:30; Fig. 2g). We predicted according to our hypothesis that the experimental group would keep their body aligned with the magnetic field as long as there was daylight. We speculate that the reason may be associated with the unnatural 2 h longer days those experimental birds experienced and that may have interfered with the birds’ internal clock. Such longer days are naturally experienced by individuals in the experimental group earlier in the season and at higher latitudes. In our experimental setup, we manipulated only the time of the sunset in experimental birds and kept the same local sunrise time unaltered for both groups. If we had time-shifted the experimental birds changing both sunrise and sunset times, we could not infer if body alignment was controlled just by the internal clock or if also daylight conditions could have a rule in the decision. A delayed sunset, on the other hand, would simulate a longitudinal displacement that birds naturally incur during days of migration. The internal clock of a bird flying across longitudes will be out of sync compared to the local time when the bird lands at the stopover location (Alerstam and Pettersson 1991). This is the reason why birds probably cannot rely only on their internal clock to perform compass calibration, but daylight information could be an important complementary cue facilitating this process.

The analysis presented above was based on pooling all the observations of the 16 individuals used in the study. Due to the non-independence of the observations, we first investigated the average of individual birds across the entire experimental period and their relative shift after sunset relative to their preferred body alignment before sunset (Supplementary Material, Fig. S2). Also in this plot we could observe the tendency of individual birds to align in the W–E magnetic direction before sunset (Fig. S2a) and turning toward a more NE–SW magnetic direction after sunset (Fig. S2b) by − 22° on average (Fig. 2Sc; see also below). However, with individual averages and a small sample size (*n* = 16) we were unable to detect any significant difference due to the substantial scatter (all tests *p* > 0.05). Hence, to handle this challenge, we decided to build a series of Bayesian circular mixed-effects models using individual ID as random intercept to account for the repeated measures of the same individual. The model comparison showed that the daylight (i.e., before or after sunset) was the best model predictor (i.e., smallest information criterions) compared to a simple random intercept model and a model using time of day as fixed effect. Furthermore, sunset time could explain 16° ± 3° of the random intercept variance and predicted a body alignment before sunset (daylight present) compatible with an E–W magnetic alignment (mean = 73°–253°, mode = 69°–249°, SD = 14°, HPD = 50°–230°/102°–282°) and a body alignment after sunset (daylight absent) rotated by 10º toward the magnetic N–S direction (mean = 64°–244°, mode = 61°–241°, SD = 10°, HPD = 48°–228°/85°–265°). The estimation of the mean direction before sunset was lower than the ideal magnetic alignment 90°–270° and it was included in the range of the after sunset HPD estimation. The lower mean estimate was probably due to the unnatural 2 h longer days experienced by the experimental group as already discussed in the paragraph above and, hence, the response to a cue-conflict between time of day and daylight that is also visible in the distributions of Fig. 2f and g. Notwithstanding such possible bias in the mean, only before sunset, the W–E geomagnetic axis was included in the 95% highest posterior density interval (shown above). Furthermore, as outlined before, the expected SW migratory direction of the species tested could contribute to the overlap of the two distributions estimated by the model for before sunset and after sunset, respectively.

Our facility is equipped with 3-dimensional Merritt coils (Ilieva et al. 2018; Bianco et al. 2019b), but we did not include in our experimental design any change of the geomagnetic field polarity for the experimental group, or the direction of any other cue by 90º as commonly done in magnetosensation or cue-conflict experiments (e.g., Sjöberg and Muheim 2016; Pakhomov and Chernetsov 2020). The outcome of such experiment could have been difficult to interpret, since many magnetic alignment studies, including the ones performed with songbirds, show bi-axial responses where animals align both parallel (0°) and orthogonal (90°) to the geomagnetic field (Begall et al. 2013; Bianco et al. 2019a). Our results, however, showed that the tested species has a magnetic E–W preference for body alignment, hence, opening for follow-up studies where the polarity of the geomagnetic field can be experimentally shifted by 90°. A response to a geomagnetic manipulation will greatly contribute to the evidence that magnetic body alignment in songbirds is timed at sunset time. Moreover, using MHz range frequency fields, local anaesthesia, or electromagnetic induction, it will be possible to disentangle whether the magnetic body alignment is based on the radical-pair or the magnetite-based mechanisms and whether the same magnetosensation process is at play during daytime and at night (Stapput et al. 2008; Wiltschko et al. 2010; Nimpf et al. 2019).

We implemented a custom machine learning framework that did not rely on fine-tuning heuristics settings as in a previous approach (Bianco et al. 2019a). In practice, the DNN used is extremely robust and allowed us to efficiently track the body alignment of 16 birds at high frequency (5 Hz) for several hours per day for 2 entire weeks, that is, for more than 20 million times. Thanks to our efficient machine learning approach, we could repeatedly measure individual birds at specific times of the day (Begall et al. 2013; Hart et al. 2013b; Bianco et al. 2019a) by pooling repeated individual measures. However, methods to analyse repeated measurements of angular data are limited and still not widespread (recently reviewed by Pewsey and García-Portugués 2021). The circular mixed-effect model implemented in the R package *bpnreg* estimates model parameters using a Bayesian approach implementing a Markov Chain Monte Carlo sampler on the projection of angular data into a bivariate linear space (Nuñez-Antonio and Gutiérrez-Peña 2014). Except for choosing a relatively high iteration number to ensure sampler convergence, the model is constructed and inspected similarly as in linear-mixed-effect models (e.g., Bates et al. 2015) that are largely used in ecology and, more specifically, to study short time series of behavioural phenotypic variation of captive birds, including examples from the same experimental facility used in this study (Ilieva et al. 2018; Bianco et al. 2019b). We anticipate that the machine learning approach outlined in this study could be easily implemented in other animal systems, and could be implemented to create a solid framework to study body alignment and the temporal pattern of this behaviour in different settings. Moreover, although large sample size is always desirable, in cases where it is difficult or not possible to use traditional circular statistics (Batschelet 1981; Landler et al. 2018, 2021), as for our migratory birds which passage is limited in a restricted seasonal migratory period, modern modelling techniques can generate robust predictions by leveraging repeated measures of a relatively small sample size (Pewsey and García-Portugués 2021). Furthermore, circular mixed-effect models will allow to make inferences in experimental settings where contrasting factors are at play, like in cue-conflict experiments, because they are also capable to handle multiple covariate predictors (Nuñez-Antonio and Gutiérrez-Peña 2014; Pewsey and García-Portugués 2021).

## Conclusions

In this study, we provide the first experimental evidence that magnetic body alignment with cardinal magnetic directions occurs in a nocturnal migratory songbird at sunset, and that such behaviour could be extended in response to an artificially delayed sunset. The time when birds align with the geomagnetic field is presumably not set by their internal clock alone, but daylight seems to be an important complementary cue (Muheim et al. 2002; Wiltschko et al. 2010). Daylight variation seems to act as an important control mechanism to compensate for drift of the internal clock during migration (Åkesson and Helm 2020) and it can trigger an immediate response after a single day of exposure (Åkesson et al. 2021a). This daylight control would be particularly important at high latitudes when longitudinal displacements are much quicker (Alerstam and Pettersson 1991). Since we observed magnetic alignment at sunset, it is possible that this behaviour may be part of the compass calibration process (Åkesson et al. 1996; Cochran et al. 2004; Muheim et al. 2006).

Birds have access to a number of different cues for compass orientation, including the Earth’s magnetic field (Wiltschko and Wiltschko 1972), the stars (Emlen 1970), the sun (Kramer 1957), and the associated pattern of skylight polarization (Able 1982). Different studies have tried to explain how each compass mechanism can translate into a migratory route, but so far, there is no conclusive answer on which compass mechanism is used during migration (Alerstam et al. 2001; Åkesson and Bianco 2016, 2017). The reason why no compass mechanisms explain observed natural migration resides in the lack of understanding of how multiple environmental cues are integrated into a single biological compass (Alerstam 2006). There have been numerus attempts to understand how compass calibration works during cue-conflict experiments (reviewed in Sjöberg and Muheim 2016, Pakhomov and Chernetsov 2020), but to the best of our knowledge, magnetic body alignment has never been considered neither in cue-conflict experiments (discussed above) nor in studies addressing the ontogeny of celestial cues in the bird’s compass (e.g., Emlen 1970; Able and Able 1996; Zolotareva et al. 2021). We suggest that the analysis of magnetic body alignment should be included in future studies aiming at understanding ontogeny and calibration of biological compasses.

During night-time, all birds in our experiment aligned along the axis of the expected migratory direction (Fig. 2). We already observed such behaviour in a diurnally migrating songbird species, the dunnock (Bianco et al. 2019a). If body alignment is involved in orientation behaviour (see Section “Results and discussion” above), we are facing a methodological shortcoming in orientation studies using Emlen-funnels (Emlen and Emlen 1966). Bianco et al. (2016) reported that there was no relationship between the body alignment direction and the orientation of European robins recorded in Emlen-funnels. In fact, the small size and the sloping walls of the funnel cage are affecting the body position of the bird after each take-off attempt. Hence, if body alignment is involved in the take-off orientation of migratory birds, the funnel itself will affect the bird’s performance, and given this interference, this might explain at least in part the large scatter commonly found in orientation experiments performed in Emlen-funnels, particularly when testing juveniles (Åkesson et al. 2021b). We suggest that future studies using Emlen-funnel experiments should carefully investigate how the shape of the funnel affects the bird’s body position, which may lead to necessary modifications such as the use of larger cages and/or steeper funnel walls (e.g., Bianco et al. 2016; Busse 2017). Thus, at this point, Emlen-funnels remain a complementary tool for orientation studies, but we argue they cannot act as a substitute for body alignment measurements.

Finally, the experimental setting presented in this study should be extended to more bird species and to other taxa to deepen our understanding of the underlying mechanisms of magnetic body alignment, its involvement in compass calibration and navigation, and its ecological and evolutionary significance in the many taxa it has been so far observed.

## Supplementary Information

Below is the link to the electronic supplementary material.

Supplementary file1 (PDF 338 KB)

## Data Availability

Data are available from the authors on request.
