# Supplementary material for: The importance of time of day for magnetic body alignment in songbirds
Source: J Comp Physiol A Neuroethol Sens Neural Behav Physiol. 2022 Jan 7;208(1):135–44. doi: 10.1007/s00359-021-01536-9 (PMC8918448; doi:10.1007/s00359-021-01536-9)
Supplement: Supplementary file 1 — Supplementary file1 (PDF 338 KB) [file 359_2021_1536_MOESM1_ESM.pdf]

The importance of time of day for body alignment in songbirds

Giuseppe Bianco, Robin Clemens Köhler, Mihaela Ilieva, Susanne Åkesson

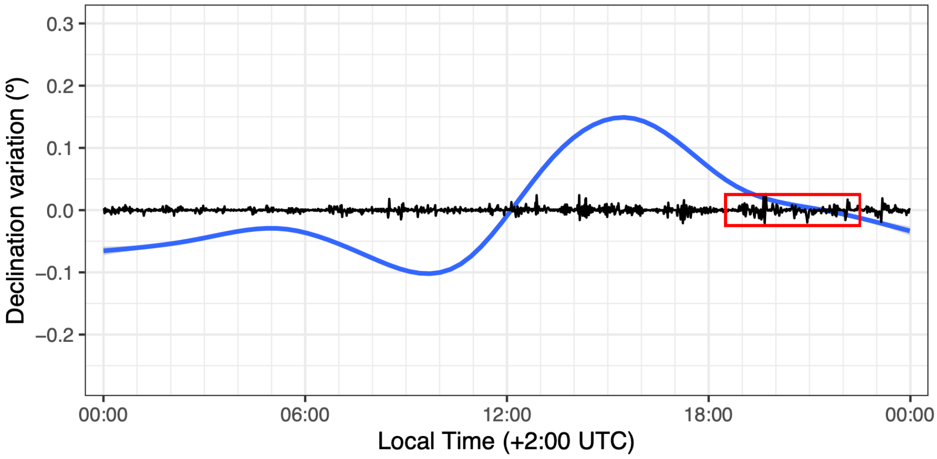

Fig. S1 Example of inspection of the geomagnetic field variation over the course of the experiment. The blue line is a local regression of declination variation around its daily mean during the entire duration of the experiment (14 days) and shows the natural daily variation of the local geomagnetic field. The black line represents the declination variation per minute during a single day. The width of the red rectangle represents the experimental time. The height of the red rectangle is arbitrarily chosen to be 0.05° to show the intensity level of high frequency variation that could potentially influence the bird's behaviour.

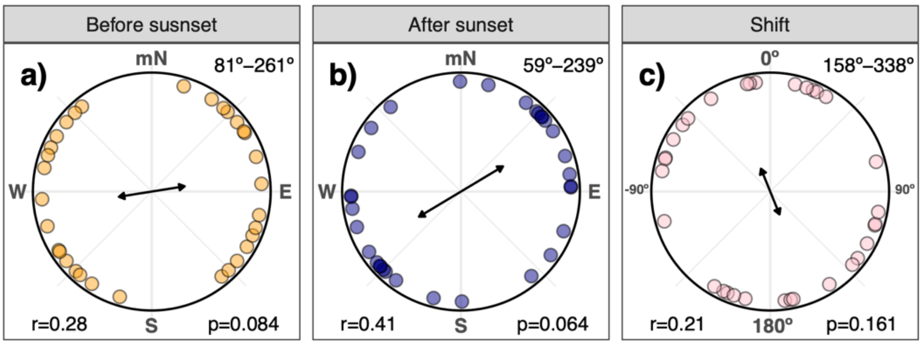

Fig. S2 Body alignment of reed warblers (*Acrocephalus scirpaceus*) measured relative to the magnetic North (mN) before natural or artificial sunset (a) or after sunset (b). Also shown is the relative shift in alignment after the sunset relative to the orientation before sunset (c). Pair of opposite dots around the circle represent the mean of body-axis for an individual bird (n=16). The double-headed arrows represent the mean vector of axial orientation with length equal to the concentration parameter (r) and drawn relative to the unitary circle radius. Rayleigh test of uniformity (p) are also reported.
